# Sex differences in readmission rate after cardiac surgery

**DOI:** 10.3389/fcvm.2023.1273785

**Published:** 2023-10-11

**Authors:** Luca Koechlin, Jules Miazza, Brigitta Gahl, David Santer, Luise Vöhringer, Denis Berdajs, Friedrich S. Eckstein, Oliver Reuthebuch

**Affiliations:** ^1^ Department of Cardiac Surgery, University Hospital Basel, Basel, Switzerland; ^2^ Surgical Outcome Research Centre Basel, University Hospital Basel, University Basel, Basel, Switzerland; ^3^ Medical Faculty of the University of Basel, Basel, Switzerland

**Keywords:** cardiac surgery, readmission, health care, sex differences, epidemiology

## Abstract

**Introduction:**

The impact of sex on hospital readmission rate after cardiac surgery is unclear. Therefore, we aimed to analyse sex-specific differences and underlying factors in 30-day readmission rate after cardiac surgery.

**Methods:**

We conducted a single center study including all patients after major cardiac surgery (excluding aortic dissection and left ventricular assist device implantation) from January 2012 to September 2020. Reasons for readmission were adjudicated according to all available medical records. We calculated incidence rate ratios (IRR) with 95% confidence intervals (CI) for female sex with re-admission crude and adjusted for plausible confounding factors using negative binomial regression.

**Results:**

4,868 patients were included in the analysis. The median [Interquartile range] age was 68 [60 to 74] years and 24% (*n* = 1,149) of the patients were female. Female patients were significantly older (median [IQR] age 70 (63 to 76) vs. 67 (59 to 74), *p* < 0.001) and had lower body mass index and fewer cardiovascular risk factors compared to men. Isolated valve surgery was more frequent in female while coronary artery bypass grafting was more often in men. 30-day readmission was comparable between both sexes (7.0% [*n* = 81] in female vs. 8.7% [*n* = 322] in men; *p* = 0.078). Cardiac related readmissions and infections were the most common reasons for readmission in both groups. The overall incidence rate ratios of female sex with readmission (0.80, 95% CI 0.63 to 1.03, *p* = 0.078) remained robust after adjustment for EuroSCORE 2 (0.78, CI 0.61 to 1.0, *p* = 0.051).

**Conclusion:**

Readmission rate and reasons for 30-day readmission after major cardiac surgery were similar between men and women.

## Graphical Abstract

Created with BioRender.com 
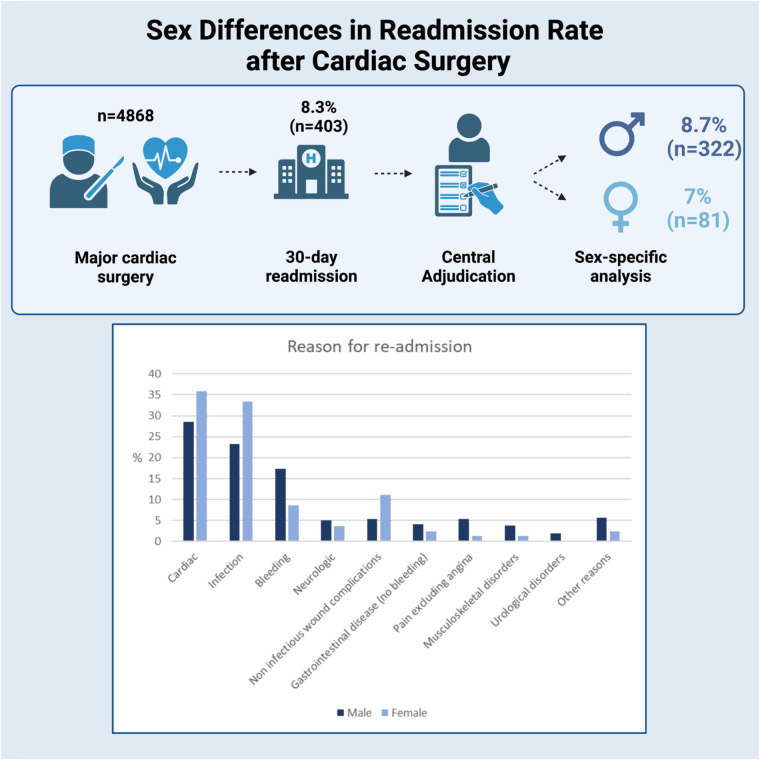

## 1. Introduction

Readmission after cardiac surgery reflects a major burden for the health-care system and imposes financial and health burdens on patients and the society (1–6).

While previous studies mainly investigated hard outcome data such as mortality, soft outcomes such as rehospitalisation or patient related outcome measures still are underrepresented in current outcome research.

Recent pilot studies investigating readmission rate and underlying factors after cardiac surgery spotted several contiguous risk factors such as increasing age, African-American race, higher body mass index, numerous comorbidities, post-operative complications and lower socioeconomic status (2–4, 7). However, regarding the impact of sex, there exist some uncertainties since either female or male sex have been previously reported as risk factor for readmission after cardiac operations (2–5, 8).

Furthermore, to the best of our knowledge, sex-specific differences regarding the underlying factors for readmission are not investigated so far. This is of particular interest since at least a part of readmissions seem to be avoidable (9).

To address this major gap in clinical knowledge, we aimed to analyse sex-specific differences in 30-day readmission rate and underlying factors.

## 2. Material and methods

### 2.1. Ethical approval

The study was conducted according to the principles of the declaration of Helsinki, approved by the local ethical committee (EKNZ BASEC Req-2020-02023) and registered at ClinicalTrials.gov (ID NCT05225246). The ethical committee has waived the need to obtain informed consent.

### 2.2. Study design

We constructed the single centre retrospective observational DREAMS (Database for REAdmission after Major cardiac Surgery) registry including all patients after major cardiac surgery from January 2012 to September 2020. Patients with minor operations such as pacemaker implantation, pericardiocentesis, or in-hospital death were excluded from this analysis. Additionally, we excluded patients with aortic dissection or left ventricular assist device since these patients represent a highly special cardiac surgical patient cohort with a high-risk for readmission. Furthermore, re-operations/revisions within the same admission were not registered twice.

Using a prospectively maintained institutional registry (Intellect 1.7, Dendrite Clinical Systems, Henley-on-Thames, United Kingdom), we identified all patients who underwent major cardiac surgery in our institution. The clinical data were exported from this registry where data have been regularly controlled for completeness and accuracy. Intraoperative data were collected prospectively in a standardized fashion. For each admission with major cardiac surgery, further referred to as index admission, we extracted data on stationary and ambulatory readmissions within 30 days after discharge from the electronic health record using the hospitals datawarehousing solution.

### 2.3. Adjudication of the cause for readmission

All readmissions to our tertiary centre within 30 days after discharge from index admission were analyzed. To maintain consistency, reasons for readmission were adjudicated by the same research fellow (J.M.) according to all available medical records obtained during clinical care including history, physical examination, results of laboratory testing, radiologic testing, electrocardiogram and echocardiography. In case of uncertainty, patient records were reviewed by two other members of the research group (L.K., O.R.).

Based on previous literature and medical considerations, causes for readmission were categorized in cardiac related, infections, bleeding, neurological, gastrointestinal disease excluding bleeding (such as gastritis, cholecystitis or diverticulitis), pain excluding angina, noninfectious wound complications, musculoskeletal disorders, urological and other (7, 10). Detailed information about the categories and the definitions is provided in Online Supplementary Table S1.

### 2.4. Statistical analysis

To investigate whether patient sex is associated with the risk of re-admission within 30 days, we calculated crude and adjusted incidence rate ratios (IRR) with 95% confidence intervals using negative binomial regression. For the adjustment, we included age, COPD, EuroSCORE 2, emergency, type of surgery (coronary artery bypass grafting, aortic valve surgery, mitral valve surgery, aortic surgery) and time of extracorporeal circulation (min) as covariates. We first included one of these covariates into the model together with sex, then we included an interaction term of the covariate and sex to check for different associations in women and men. Lastly, we included all variables. To give an overview of the association of sex and risk of re-admission, we showed all IRRs in a forest plot. We visualized time to first re-admission using Kaplan-Meier plots. Moreover, we conducted four subgroup analysis. First, we focussed on type of surgery, e.g. isolated coronary artery bypass grafting, isolated aortic valve surgery, and isolated mitral valve surgery, then we derived risk of readmission by type of cardiac rehabilitation (in-patient vs. out-patient) as a sensitivity analysis.

Continuous variables were shown as median with interquartile range and compared using Wilcoxon-Mann-Whitney test. Categorical variables were shown as number with percentage and tested using Fisher's exact test. All analyses were carried out by a biostatistician (B.G.) using Stata 16 (Stata Corp., College Station, Texas).

## 3. Results

### 3.1. Study cohort

A total of 5,512 standard cardiac operations were carried out in our centre between January 2012 and September 2020. After exclusion of surgery for aortic dissection (*n* = 295), left ventricular assist device implantation (*n* = 16), minor procedures (*n* = 116), consecutive operations within the same admission (*n* = 42) and in hospital death (*n* = 175), the final study cohort comprised a total of 4,868 patients, 1,149 patients (24%) were female. Median [Interquartile range (IQR)] age was 68 (60 to 74) years, while female patients were significantly older compared to male patients (70 [63 to 76] vs. 67 [59 to 74] years; *p* < 0.001). Isolated valve surgery, e.g., single, double or triple valve procedure without further concomitant procedures, was more frequent in female [41% (*n* = 474) vs 22% (*n* = 815)] while coronary artery bypass grafting was more often performed in male (27% [*n* = 305] vs. 46% [*n* = 1,700]). Baseline characteristics and procedure related information are depicted in Tables 1, 2 and postoperative data in the Online Supplementary Table S2.

**Table 1:** Patient characteristics by gender.

	Total (*N* = 4,868)	Male (*N* = 3,719)	Female (*N* = 1,149)	*p*
Age, years	68 (60 to 74)	67 (59 to 74)	70 (63 to 76)	<0.001
BMI, kg/m²	26 (24 to 29)	27 (24 to 29)	25 (22 to 30)	<0.001
Diabetes Mellitus				0.023
No	3,717 (76%)	2,804 (75%)	913 (79%)	
Diet	169 (3.5%)	136 (3.7%)	33 (2.9%)	
On oral antidiabetics	574 (12%)	463 (12%)	111 (10%)	
Insulin	408 (8.4%)	316 (8.5%)	92 (8.0%)	
Hypertension	3,825 (79%)	2,947 (79%)	878 (76%)	0.044
Hypercholesteremia	2,996 (62%)	2,373 (64%)	623 (54%)	<0.001
Current Smoker	1,060 (22%)	845 (23%)	215 (19%)	0.004
Peripheral artery disease	490 (10%)	377 (10%)	113 (10%)	0.8
Preoperative stroke	473 (10%)	360 (10%)	113 (10%)	0.9
Renal disease	292 (6.0%)	241 (6.5%)	51 (4.4%)	0.010
Last pre-operative creatinine, µmol/l	83 (71 to 99)	85 (74 to 101)	72 (61 to 88)	<0.001
Dialysis	56 (1.2%)	45 (1.2%)	11 (0.96%)	0.6
COPD	529 (11%)	400 (11%)	129 (11%)	0.7
Prior MI	1,636 (34%)	1,371 (37%)	265 (23%)	<0.001
Three vessel CAD	2,231 (46%)	1,897 (51%)	334 (29%)	<0.001
Left main CAD	666 (14%)	573 (15%)	93 (8.1%)	<0.001
NYHA				
n/a	506 (10%)	425 (11%)	81 (7.0%)	
I	1,144 (24%)	957 (26%)	187 (16%)	
II	1,702 (35%)	1,312 (35%)	390 (34%)	
III	1,266 (26%)	840 (23%)	426 (37%)	
IV	250 (5.1%)	185 (5.0%)	65 (5.7%)	
NYHA III or IV	1,516 (31%)	1,025 (28%)	491 (43%)	<0.001
AF preop	540 (11%)	394 (11%)	146 (13%)	0.053
Ejection fraction	57 (49 to 62)	56 (48 to 61)	60 (51 to 65)	<0.001
EuroSCORE 2	2.0 (1.1 to 4.2)	1.8 (1.0 to 3.7)	2.8 (1.5 to 5.7)	<0.001

BMI, body mass index; DM, diabetes mellitus; COPD, chronic obstructive pulmonary disorder; MI, myocardial infarction; CAD, coronary artery disease; NYHA, New York heart association; AF, atrial fibrillation.

**Table 2:** Operative data.

	Total (*N* = 4,868)	Male (*N* = 3,719)	Female (*N* = 1,149)	*p*
Procedure groups				<0.001
CABG and Other	177 (3.6%)	149 (4.0%)	28 (2.4%)	
CABG and Valve (s)	658 (14%)	515 (14%)	143 (12%)	
CABG and Valve (s) and other	181 (3.7%)	143 (3.8%)	38 (3.3%)	
CABG only	2,005 (41%)	1,700 (46%)	305 (27%)	
Other	67 (1.4%)	39 (1.0%)	28 (2.4%)	
Valve (s) and Other	491 (10%)	358 (10%)	133 (12%)	
Valve (s) only	1,289 (26%)	815 (22%)	474 (41%)	
OPCABG	413 (8.5%)	295 (7.9%)	118 (10%)	0.015
CABG only	2,005 (41%)	1,700 (46%)	305 (27%)	<0.001
CABG	3,021 (62%)	2,507 (67%)	514 (45%)	<0.001
Number of distal anastomoses	3.0 (2.0 to 4.0)	3.0 (3.0 to 4.0)	3.0 (2.0 to 4.0)	<0.001
Isolated aortic valve	695 (13%)	452 (12%)	243 (21%)	<0.001
Aortic valve procedure				<0.001
AV-Reconstruction*	45 (0.92%)	31 (0.83%)	14 (1.2%)	
Other	84 (1.7%)	45 (1.2%)	39 (3.4%)	
Replacement	1,449 (30%)	1,068 (29%)	381 (33%)	
Repair	96 (2.0%)	81 (2.2%)	15 (1.3%)	
TAVI	92 (1.9%)	55 (1.5%)	37 (3.2%)	
Composite graft	155 (3.2%)	132 (3.5%)	23 (2.0%)	0.009
Isolated mitral valve	464 (10%)	283 (7.6%)	181 (16%)	<0.001
Any MV	994 (20%)	653 (18%)	341 (30%)	<0.001
Mitral valve	748 (15%)	492 (13%)	256 (22%)	<0.001
Mitral valve procedure				<0.001
Replacement	244 (5.0%)	126 (3.4%)	118 (10%)	
Repair	738 (15%)	521 (14%)	217 (19%)	
Other	12 (0.25%)	6 (0.16%)	6 (0.52%)	
Isolated double/triple valve	122 (2.5%)	77 (2.1%)	45 (3.9%)	0.001
Tricuspid and/or Pulmonary valve	120 (2.5%)	69 (1.9%)	51 (4.4%)	<0.001
Tricuspid or Pulmonary valve	26 (0.53%)	14 (0.38%)	12 (1.0%)	0.010
Aortic procedure	517 (11%)	398 (11%)	119 (10%)	0.8
Emergency	299 (6.1%)	243 (6.5%)	56 (4.9%)	0.041
Operation time	210 (165 to 249)	210 (173 to 251)	195 (150 to 240)	<0.001
Perfusion time, min	107 (83 to 140)	108 (84 to 140)	105 (81 to 138)	0.066
Aortic clamping time, min	72 (54 to 96)	73 (54 to 98)	70 (52 to 91)	0.003

* With Autologous pericard.

CABG, coronary artery bypass grafting; OPCABG Off, pump coronary artery bypass grgfting; AV, aortic valve; TAVI, transcatheter aortic valve implantation; MV, mitral valve.

### 3.2. Frequency of readmission

**Figure 1:**
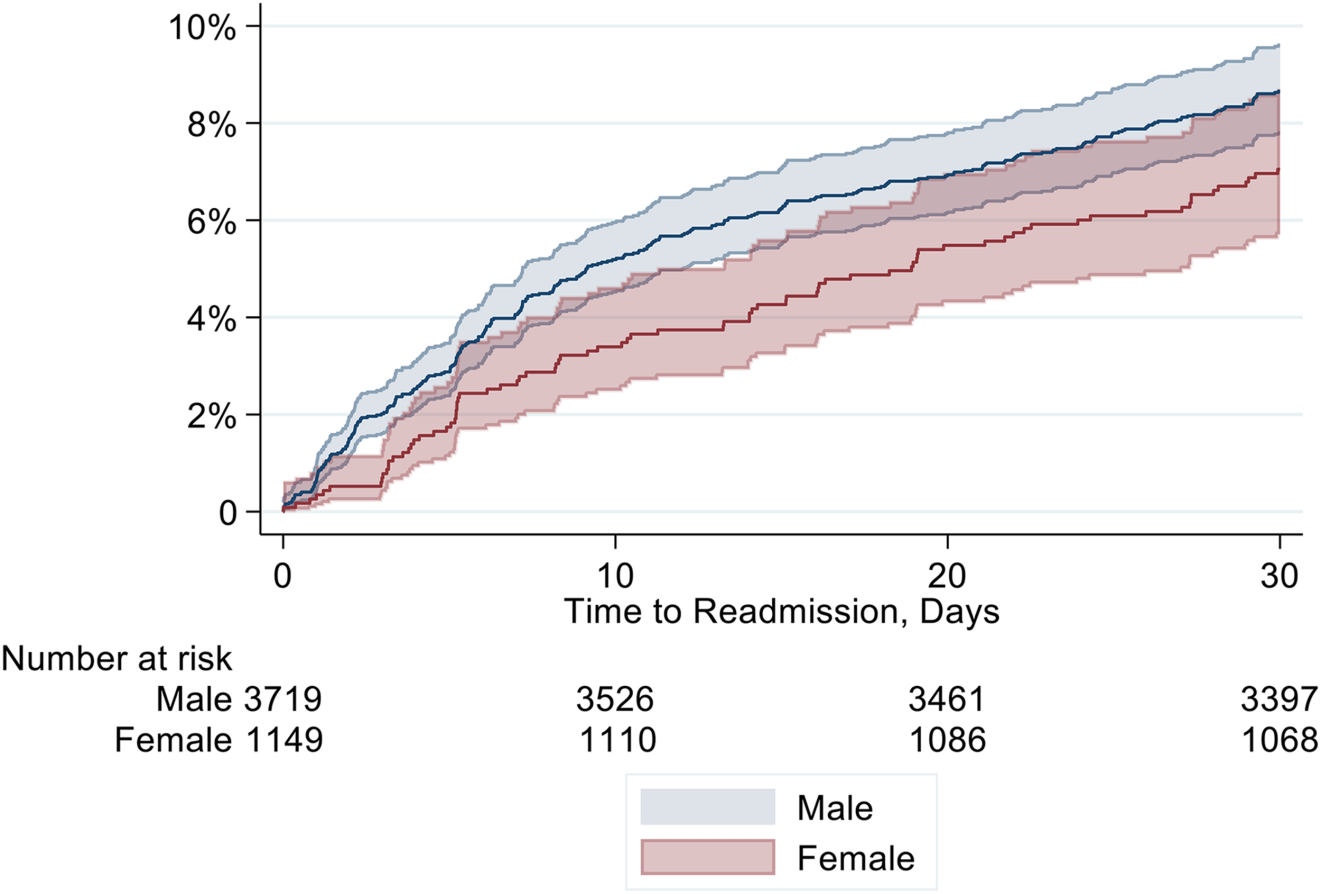
Cumulative incidence for 30-day readmission according to sex.

In the entire study cohort, frequency of readmissions within 30 days after discharge was 8.3% (*n* = 403), 7.0% (*n* = 81) in women compared to 8.7% (*n* = 322) for men. Six women (0.52%) and 31 men (0.83%) were re-admitted twice, one woman (0.09%) and three men (0.08%) were re-admitted three times, hence 444 readmissions in total, 356 in men and 88 in women. This corresponds to a readmission rate of 9.2 (8.4 to 10.1) per 100 patients within 30 days and an IRR of 0.8 (95% CI 0.63 to 1.03, *p* = 0.078) of female gender. Figure 1 displays the cumulative incidence curves for 30-day readmission.

### 3.3. Patient characteristics of readmitted patients

In male patients, readmitted patients significantly more often were current smokers and had renal disease. In female patients, only preoperative NYHA class III or IV was significantly different between the groups with higher values in patients which were readmitted. A more detailed depiction of readmitted patients for both cohorts is provided in Online Supplementary Table S3.

### 3.4. Causes for readmission

**Figure 2:**
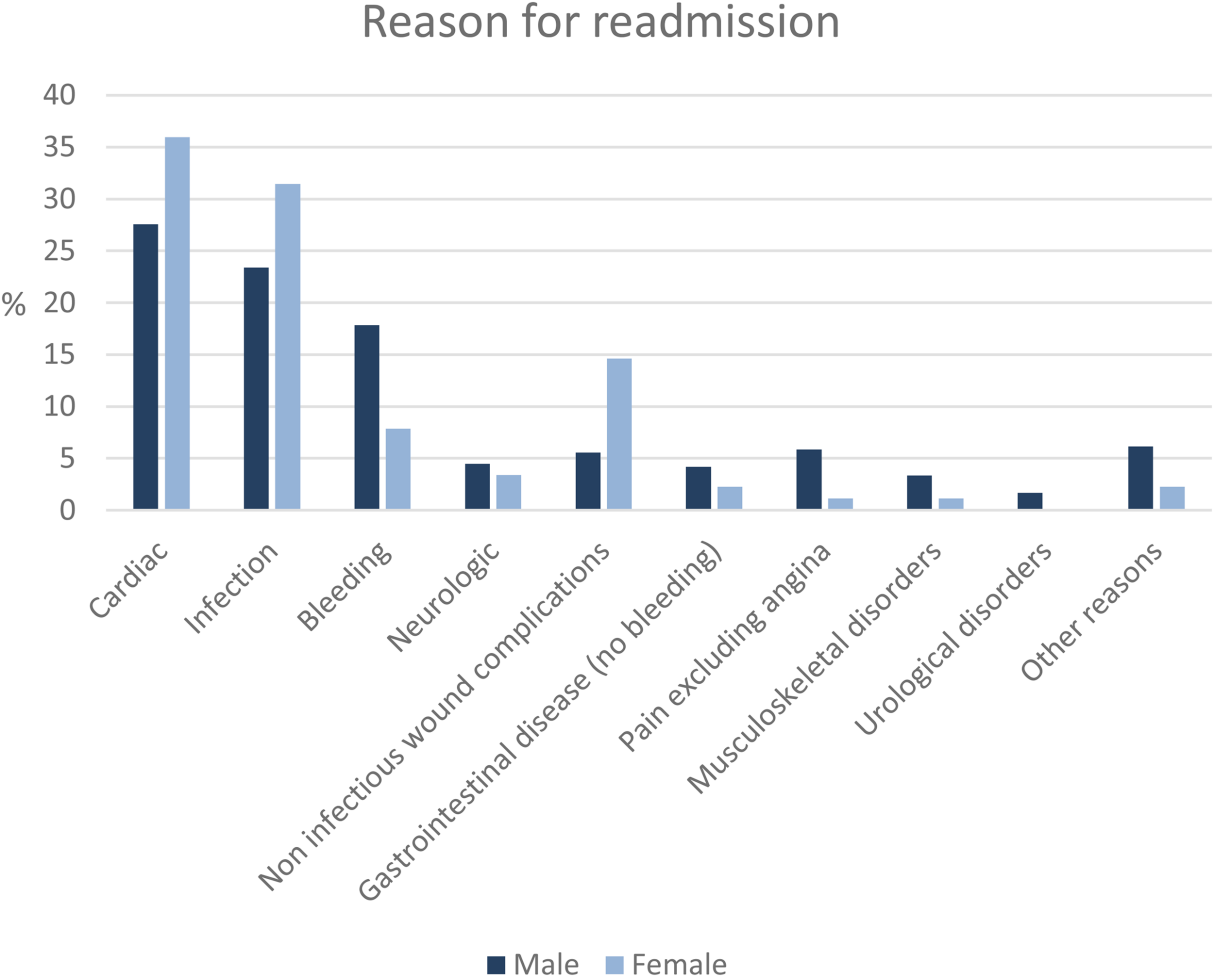
Sex-specific reasons for readmission.

The main reasons for readmission within 30 days were cardiac related and infectious (female: 36% [*n* = 32] and 31% [*n* = 28], male: 28% [*n* = 99] and 23% [*n* = 84]; respectively, *p* = 0.12 and *p* = 0.13). While bleeding complications were seen twice as often in men than in female (18% [*n* = 64] vs. 7.9% [*n* = 7], *p* = 0.023), non-infectious wound complications were 15% (*n* = 13) in female and 5.6% (*n* = 20) in men (*p* = 0.006). Detailed information about the frequency of specific reasons for readmission are provided in Figure 2 and Table 3.

**Table 3:** Reasons for readmission.

	Total (*N* = 4,868)	Male (*N* = 3,719)	Female (*N* = 1,149)	*p*
Readmission	403 (8.3%)	322 (8.7%)	81 (7.0%)	0.078*
Cardiac related	131 (29%)	99 (28%)	32 (36%)	0.12
Volume overload	38 (8.5%)	30 (8.4%)	8 (9.0%)	
Arrhythmia	35 (7.8%)	25 (7.0%)	10 (11%)	
Angina or myocardial infarction	8 (1.8%)	7 (1.9%)	1 (1.1%)	
Adjustment of anticoagulation agents	2 (0.45%)	2 (0.56%)	0 (0.0%)	
Blood pressure management	2 (0.45%)	2 (0.56%)	0 (0.0%)	
Thromboembolism	7 (1.6%)	6 (1.7%)	1 (1.1%)	
Blood glucose or electrolyte management	2 (0.45%)	0 (0.0%)	2 (2.2%)	
Dressler syndrome	30 (6.7%)	23 (6.4%)	7 (7.9%)	
Cardiac related other	7 (1.6%)	4 (1.1%)	3 (3.4%)	
Infection	112 (25%)	84 (23%)	28 (31%)	0.13
Infection in operation site	66 (15%)	49 (14%)	17 (19%)	
Infection not in operation site	32 (7.1%)	25 (7.0%)	7 (7.9%)	
Fever without identified infection source	10 (2.2%)	6 (1.7%)	4 (4.5%)	
Infection other	4 (0.89%)	4 (1.1%)	0 (0.0%)	
Bleeding	71 (16%)	64 (18%)	7 (7.9%)	0.023
Gastrointestinal bleeding	10 (2.2%)	9 (2.5%)	1 (1.1%)	
Anemia	8 (1.8%)	7 (1.9%)	1 (1.1%)	
Bleeding complication in operation site	29 (6.5%)	25 (7.0%)	4 (4.5%)	
Bleeding not in operation site	24 (5.4%)	23 (6.4%)	1 (1.1%)	
Neurological	19 (4.2%)	16 (4.5%)	3 (3.4%)	1
Cerebrovascular accident	4 (0.89%)	4 (1.1%)	0 (0.0%)	
Fall/syncope/presyncope	7 (1.6%)	6 (1.7%)	1 (1.1%)	
Neurological other	8 (1.8%)	6 (1.7%)	2 (2.2%)	
Diverse	115 (26%)	96 (27%)	19 (21%)	0.3
Non infectious wound complications	33 (7.4%)	20 (5.6%)	13 (15%)	0.006
Gastrointestinal disease (no bleeding)	17 (3.8%)	15 (4.2%)	2 (2.2%)	0.5
Pain excluding angina	22 (4.9%)	21 (5.8%)	1 (1.1%)	0.10
Musculoskeletal disorders	13 (2.9%)	12 (3.3%)	1 (1.1%)	0.5
Urological disorders	6 (1.3%)	6 (1.7%)	0 (0.0%)	0.6
Other reasons	24 (5.4%)	22 (6.1%)	2 (2.2%)	0.19

* Derived from IRR.

Note that numbers in the first row are refer to patients, other rows refer to readmissions.

### 3.5. Predictors for readmission

**Figure 3:**
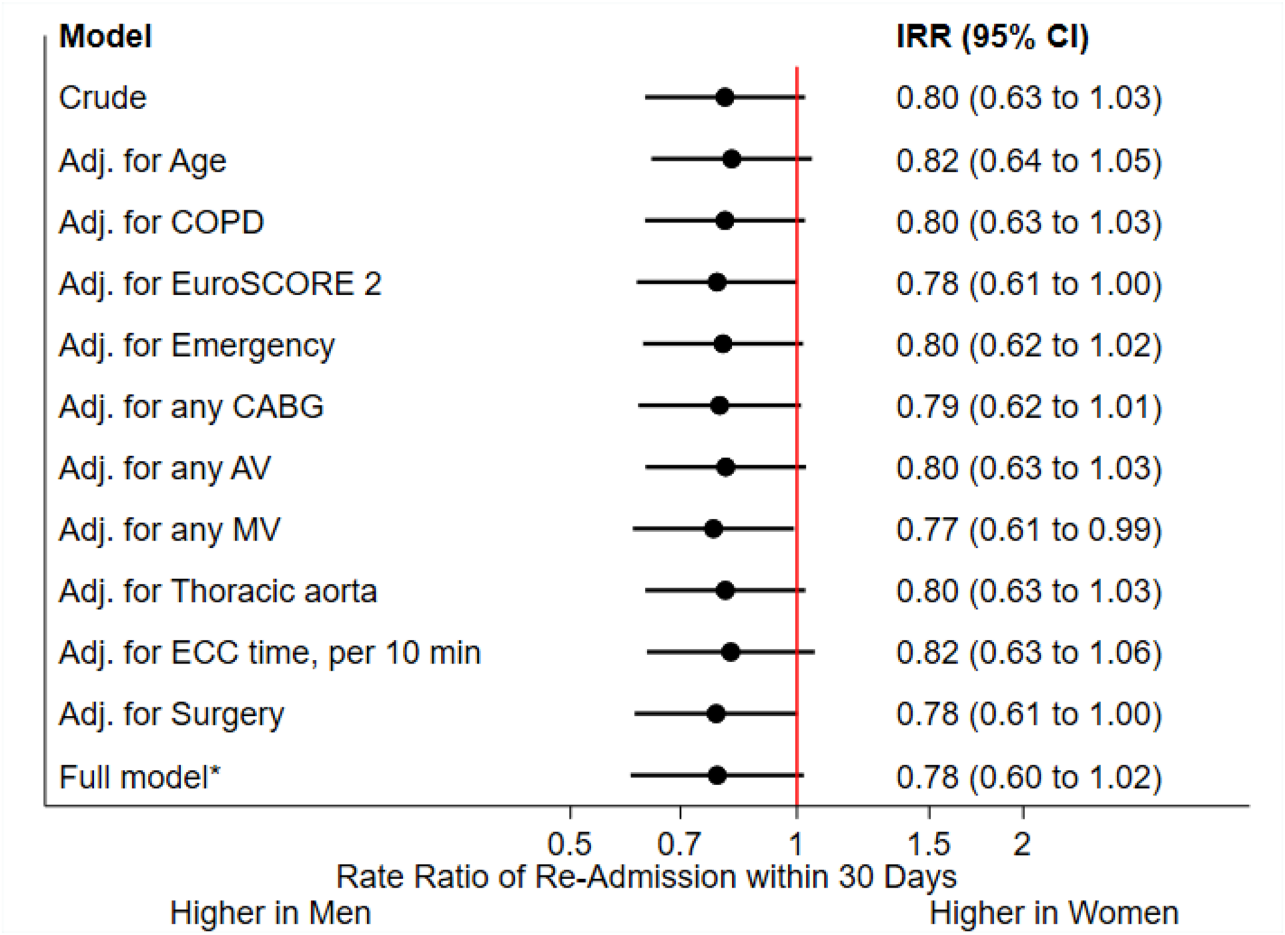
COPD, chronic obstructive pulmonary disease; CABG, coronary artery bypass grafting; AV, aortic valve; MV mitral valve; ECC, extracorporal circulation. *comprising age, COPD, EuroSCORE 2, emergency, type of surgery and ECC time.

We checked aforementioned variables for association with the risk of readmission. We found a slightly increased readmission rate with EuroSCORE II increase (IRR 1.01, CI 1.00 to 1.03, *p* = 0.012), mitral valve surgery (IRR 1.3, CI 1.03 to 1.63, *p* = 0.025) and ECC time increase (IRR 1.04 per 10 min, CI 1.02 to 1.06, *p* < 0.001) and a slightly decreased rate with age increase (IRR 0.92, CI 0.84 to 1.00, *p* = 0.047). We did not see an association with COPD, CABG, aortic valve surgery, and aortic surgery. Moreover, patients visiting an inpatient cardiac rehabilitation after the operation had a reduced risk for readmission (IRR 0.53, CI 0.39 to 0.71, *p* < 0.001). An overview is provided in Table 4 and Figure 3, as well as in Online Supplementary Table S4. Female sex did not show a significant interaction with any predictor, so we did not find evidence that associations differ between women and men.

**Table 4:** Predictors for readmission.

Variables	IRR (95% CI)	*p*
Female	0.80 (0.63 to 1.03)	0.078
Age per 10 years	0.92 (0.84 to 1.00)	0.047
COPD	1.03 (0.75 to 1.41)	0.9
EuroSCORE II	1.01 (1.00 to 1.03)	0.012
Emergency	0.64 (0.39 to 1.05)	0.075
Any CABG	0.96 (0.79 to 1.18)	0.7
Any AV	0.96 (0.78 to 1.18)	0.7
Any MV	1.30 (1.03 to 1.63)	0.025
Thoracic aorta	1.11 (0.81 to 1.51)	0.5
ECC time, per 10 min	1.04 (1.02 to 1.06)	<0.001
Inpatient Rehab^*^	0.53 (0.39 to 0.71)	<0.001

Concerning the association of complications and readmissions, all complications were associated with increased incidence of readmission. Sternal infection (IRR 5.35), sepsis (IRR 2.83) and reoperation for bleeding (IRR 1.88) showed the strongest association. However, we did not observe substantial changes of the IRR of sex. (Online Supplementary Table S5).

### 3.6. Subgroup analyses in patients with isolated CABG surgery

In 2005 patients with isolated CABG surgery (85% male [*n* = 1,700], 15% female [*n* = 305]) readmission rate was 8.0 (CI 6.9 to 9.4) per 100 patient years, and therefore slightly lower than in the entire cohort. Again, readmission frequency of male and female patients were similar (7.2% men, 122 patients with 139 readmissions vs. 6.6% women, 20 patients with 22 readmissions, *p* = 0.81). Most common reason for readmission in male patients was cardiac related [*n* = 44 (32%)] while in female patients, infection was the most frequent reason [*n* = 12 (55%); Online Supplementary Table S6, S7].

### 3.7. Subgroup analysis in patients with isolated aortic valve surgery

In 695 patients with isolated aortic valve surgery (65% male [*n* = 452], 35% female [*n* = 243]) readmission rate was 8.6 (6.7 to 11.1) per 100 patient years, concordant with the entire cohort. As in the entire cohort, readmission frequency of readmission for male and female patient was similar (9.3% [*n* = 42] male and 6.6% [*n* = 16] female, rate ratio 0.74 (95% CI 0.4 to 1.3) and *p* = 0.28). In this subgroup the main reason for readmission were cardiac related (26% [*n* = 11] male and 25% [*n* = 4] female, *p* = 1.00) and infections (26% [*n* = 11] male and 25% [*n* = 4] female, *p* = 1.00). Online Supplementary Tables S8, S9.

### 3.8. Subgroup analysis in patients with isolated mitral valve surgery

In 464 patients with isolated mitral valve surgery (61% male [*n* = 283], 39% female [*n* = 181]) overall readmission rate was 0.74 (0.4 to 1.3) per 100 patient years. Male patients were readmitted more often than female patients (11.3% [*n* = 32] vs. 4.46% [*n* = 8], with a rate ratio 0.74 (95% CI 0.4 to 1.3), *p* = 0.014). The main cause for readmission in the male cohort were infection [28% (*n* = 9)], followed by cardiac related [22% (*n* = 7)]. In the female cohort, the main cause for readmission were cardiac related [38% (*n* = 3)] followed by equal rate of readmission for infection, bleeding, gastrointestinal disease, muskuloskelettal disorder and others [each 13% (*n* = 1)]. Online Supplementary Tables S10, S11.

### 3.9. Subgroup analysis in patients with inpatient vs. outpatient cardiac rehabilitation after surgery

In patients with available data [*n* = 1,646, (34%)] concerning the type of postoperative cardiac rehabilitation (inpatient vs. outpatient), a subgroup analysis was performed (Online Supplementary Table S1^2^). Patients undergoing inpatient rehabilitation were older (69 (61 to 75) years vs. 63 (56 to 70) years; *p* < 0.001), had higher preoperative EuroSCORE II (2.0 (1.1 to 4.2) % vs. 1.3 (0.79 to 2.4) %; *p* < 0.001), as well as a higher number of comorbidities. The subgroup analysis showed that inpatient rehabilitation was associated with a lower risk of readmission [IRR 0.53 (0.39 to 0.71), *p* < 0.001]. This reduction remained unchanged when patient sex was included into the model as covariate.

## 4. Discussion

This study was conducted to analyse sex-specific differences regarding readmission rate and underlying factors for readmissions after major cardiac surgery. We notify six major findings.

First, although there was a trend to lower readmission frequency in female patients after major cardiac surgery, there was no statistically significant difference [female: 7% (*n* = 81)] vs. men 8.7% [*n* = 322], *p* = 0.078). This finding was confirmed when adjusted for confounders. Second, while readmitted male patients were more often current smokers and had renal disease, in female patients only preoperative NYHA class differed between readmitted and non-readmitted patients. Third, in both sexes, cardiac related and infection were the main reasons for readmission within 30 days. In male patient, the third most common cause for readmission were bleedings, while it was non-infectious wound complications in female. Fourth, predictors for readmission were similar for male and female as no adjustment modified the association of sex and readmission. We found slightly increased readmission rate with EuroSCORE II increase, mitral valve surgery and extracorporeal circulation time increase and a slightly decreased rate with age increase. While all complications were associated with increased incidence of readmission, including sex did not alter these results and there were no substantial changes of the IRR of sex. Furthermore, patients visiting an inpatient cardiac rehabilitation after the operation had a reduced risk for readmission. Fifth, in the subgroup analysis of patients with isolated CABG surgery, as well as in patient with isolated aortic valve surgery, similar findings were seen. Sixth, in the subgroup analysis of patient undergoing isolated mitral valve surgery, a higher rate of readmission was observed in male patients. Furthermore, the main cause for readmission in male patients was infection, which diverged from the entire cohort.

These findings extend and corroborate previous research on the topic of readmission rate after cardiac surgery. The overall readmission rate of 9.2 (CI 8.4 to 10.1) and 8.0 (CI 6.9 to 9.4) in the subgroup of isolated CABG per 100 patient years was lower compared to previously reported 30 days readmission rates (2–4, 7). Furthermore, there was no significant difference regarding readmission rate between female and male patients. While numerous studies focused on readmission rate after cardiac surgery, especially after CABG or valve surgery, to the best of our knowledge this is the first study evaluating sex-specific differences after major cardiac surgery.

Although there is rising evidence that female patients have poor outcomes after cardiac surgery, regarding readmissions, the existing literature so far still is contradictory (11, 12). While female sex was found to be associated with higher readmission rates in several studies, other studies showed no difference between male and female patients, especially, when adjusted for disease severity (2–4, 8, 10). Most of these studies evaluated readmission rates after CABG surgery. In our cohort, we did not find a statistically significant difference in the overall cohort and neither in the subgroup analysis only including patients after CABG surgery. Similar findings were observed in the subgroup analysis of patient undergoing isolated aortic valve surgery. In a subgroup analysis of patient undergoing isolated mitral valve surgery, however, higher rate of readmissions were observed in the male cohort. In this study, there were no differences regarding the reasons for readmission with cardiac related followed by infections being the most common reason for readmission in both sexes. The third most common cause for readmission was different between male and female (bleeding vs. non-infectious wound complications). Although there was no overall difference in readmission rate, differences in reasons for readmission are important for individualized medicine approaches. E.g., careful assessment of male patients' anticoagulation and bleeding risk, as well as careful wound inspection and securing condition for optimal wound healing in female patients are potential working points. However, our findings support in general the finding, that there do not seem to be significant sex-specific differences regarding readmission after cardiac surgery. Reasons for this and the at least in part contradictory findings compared to previous studies (mostly from the U.S.) may be various including a very high developed health-care system in Switzerland as well as a high general socioeconomic status of the population living in Switzerland.

Another finding of this study is the decreased risk for readmission in patients participating in an inpatient cardiac rehabilitation program after surgery. Reasons to discharge a patient to an inpatient cardiac rehabilitation facility generally are older age, higher number of comorbidities or reduced autonomy. While the risk profile of patients in an inpatient rehabilitation generally is higher, it is possible that continuous assessment by the on-site physician prevent readmission for the management of some postoperative complications. The influence of cardiac rehabilitation on 30-day readmission should therefore be addressed in further studies.

Further interesting findings include the significantly higher rate of bleedings as reason for readmission in male patients and the significantly higher rate of non-infectious wound complications in female patients. Further studies with respective design need to investigate these findings in more detail to elaborate potential underlying patient and/or procedure related factors for these findings.

Some limitations must be considered when interpreting the above-mentioned findings. First, this was a single-centre study in Switzerland and therefore other studies in other health-care systems are warranted to confirm our findings. Second, only readmissions referred to our centre were included in this study. Therefore, the rate of readmission may be underestimated in case patients were referred to other hospitals. However, since we are the only centre for cardiac surgery in our area and patients are generally readmitted to the referring hospital, it is presumable, that this might encompass only a small number of patients. Furthermore, we consider it unlikely, that there is a statistically different proportion of male and female patients regarding allocation influencing the conclusion of this analysis. Third, data concerning the type of cardiac rehabilitation underwent by patient after surgery were only available starting July 2017, thus limiting the accuracy of the subgroup analysis.

## 5. Conclusion

In our study cohort, readmission rate and reasons for 30-day readmission after major cardiac surgery were similar between men and women. Furthermore, patients undergoing mitral valve surgery have a slightly higher rate of 30-day readmission after cardiac surgery, as well as patient visiting an outpatient cardiac rehabilitation. Further studies are warranted to analyse the underlying factor for these results.

## Data Availability

The datasets presented in this article are not readily available based on ethical restrictions. We do not provide our dataset without reasonable request. Requests to access the datasets should be directed to oliver.reuthebuch@usb.ch.
