# Supplementary material for: Sex differences in readmission rate after cardiac surgery
Source: Front Cardiovasc Med. 2023 Oct 11;10:1273785. doi: 10.3389/fcvm.2023.1273785 (PMC10598725; doi:10.3389/fcvm.2023.1273785)
Supplement: Supplementary file 1 [file Datasheet1.docx]

Sex Differences in Readmission Rate after Cardiac Surgery - Supplementary Material

# Supplementary Figures and Tables

## Table 1 Reasons for readmission

|  | **Main diagnoses for readmission** | |
| --- | --- | --- |
| **Cardiac related** | Angina or myocardial infarction | Myocardial infarction  Chest pain if therapy for angina was started or indication for ischemia diagnostic |
|  | Arrhythmia | Atrial fibrillation  Atrial flutter  Ventricular fibrillation  Extrasystole |
|  | Blood pressure management |  |
|  | Dressler syndrome | Clinical manifestation of Dressler syndrome (chest pain, pericardial effusion, symptom improvement with NSAR and Colchicin)  Pericardial effusion without indication of haematoma or hemorrhage.  Pleural effusion with concomitant, hemodynamically relevant, not hemorrhagic, pericardial effusion |
|  | Volume overload (including pleural effusions requiring diuresis; thoracentesis; or tube thoracostomy) | Cardiac decompensation (dyspnea, edema, pleural effusion)  Symptoms improving with diuretics  Pleural effusion needing punction with liquid of serous consistency |
|  | Adjustment of anticoagulation agents |  |
|  | Thromboembolism | Valve thrombosis  Deep vein thrombosis  Pulmonary embolism |
|  | Blood glucose or electrolyte management |  |
| **Infections** | Infection in operation site | Wound infection sternum  Wound infection leg/arm after vein/artery harvesting for CABG  Wound infection groin after cannulation  Infective endocarditis  Pacemaker associated infection  Bacteremia if patient under antibiotics for infective endocarditis  Pleural empyema if the pleural space was opened for the operation (i.e. CABG) |
|  | Infection not in operation site | Every infection not in operation site, heart or lung (only if the pleural space was **not** open for the index operation). |
|  | Fever without an identified source of infection |  |
| **Bleedings** | Bleeding complication in operation site | Bloody pericardial effusion  Bleeding after vein/artery harvesting for CABG  Hemothorax after anterolateral access for surgery |
|  | Bleeding complication not in operation site | Every bleeding not in operation site  **Including**: Bleeding that may have been precipitated by the anticoagulation (i.e. macrohematuria) |
|  | Gastrointestinal bleeding | Upper GI Bleeding  Lower GI Bleeding  Melaena |
|  | Anemia |  |
| **Neurological** | Cerebrovascular accident | Stroke (hemorrhagic/ischemic) |
|  | Fall/syncope/presyncope |  |
|  | Neurological Others | Epilepsy  Critical illness polyneuropathy  Guillain-Barré Syndrome  Transient global amnesia  Confusion  Paresthesia  Spinal liquor fistula  Desorientation |
| **Gastrointestinal disease excluding bleeding** | Gastrointestinal disease excluding bleeding | Abdominal pain (only if no abdominal operation was performed during index hospitalization, i.e. abdominal aorta)  Gastritis  Cholecystitis  Diverticulitis  Pancreatitis  Inguinal herniation  Drug induced hepatitis |
| **Pain excluding angina** | Pain excluding angina | Chest pain (i.e. musculoskeletal)  Pain in operation site (vein harvesting, groin after cannulation)  **Excluded:** Trauma, pain without direct relation with the operation (i.e. headache) |
| **Noninfectious wound complications** | Noninfectious wound complications | Lymphocele of inguinal/femoral region after cannulation Lymph Fistula of inguinal/femoral region after cannulation  Wound dehiscence without putrid secretion and without increase in inflammatory parameters  Keloid |
| **Musculoskeletal disorders** | Musculoskeletal disorders | Arthrosis  Gout  Bursitis  Trauma  Pain of musculoskeletal origin **if not** directly related to the operation |
| **Urological** | Urological | Incontinence  Prostate hyperplasia  Paraphimosis  Complication following urological procedure  **Excluded:** Urinary tract infections |
| **Other reasons** | Other reasons |  |

## Table 2 Postoperative data

|  | Total  (N = 4868) | Male  (N = 3719) | Female  (N = 1149) | p |
| --- | --- | --- | --- | --- |
| Length of ICU stay, days | 2.0 (1.0 to 3.0) | 2.0 (1.0 to 3.0) | 2.0 (1.0 to 3.0) | <0.001 |
| Intubation >72h | 114 (2.3%) | 83 (2.2%) | 31 (2.7%) | 0.4 |
| Reoperation for bleeding | 191 (3.9%) | 147 (4.0%) | 44 (3.8%) | 0.9 |
| Postoperative MI | 77 (1.6%) | 59 (1.6%) | 18 (1.6%) | 1 |
| Postoperative Stroke | 173 (3.6%) | 121 (3.3%) | 52 (4.5%) | 0.045 |
| AF at discharge | 1346 (28%) | 990 (27%) | 356 (31%) | 0.004 |
| Permanent pacemaker | 248 (5.1%) | 179 (4.8%) | 69 (6.0%) | 0.12 |
| Sternal infection | 102 (2.1%) | 70 (1.9%) | 32 (2.8%) | 0.076 |
| Postoperative renal failure | 192 (3.9%) | 143 (3.8%) | 49 (4.3%) | 0.5 |
| Renal substitution therapy | 60 (1.2%) | 45 (1.2%) | 15 (1.3%) | 0.8 |
| Pulmonary infection | 314 (6.5%) | 249 (6.7%) | 65 (5.7%) | 0.2 |
| MACCE | 238 (4.9%) | 174 (4.7%) | 64 (5.6%) | 0.2 |
| Sepsis | 75 (1.5%) | 62 (1.7%) | 13 (1.1%) | 0.2 |
| Length of stay | 8.0 (7.0 to 11) | 8.0 (7.0 to 11) | 9.0 (7.0 to 13) | <0.001 |
| *ICU* intensive care unit; *MI* myocardial infarction; *AF* atrial fibrillation; *MACCE* major adverse cardiac or cerebrovascular event | | | | |

## Table 3 Patient characteristics by gender and outcome

|  | Females | | |  | Males | | |
| --- | --- | --- | --- | --- | --- | --- | --- |
|  | Not readmitted  (N = 1068) | Readmitted (N = 81) | p |  | Not readmitted  (N = 3397) | Readmitted  (N = 322) | p |
| Age | 70 (63 to 76) | 70 (60 to 75) | 0.4 |  | 67 (59 to 74) | 67 (58 to 74) | 0.6 |
| BMI, kg/m² | 25 (22 to 29) | 27 (22 to 31) | 0.15 |  | 27 (24 to 29) | 26 (24 to 29) | 0.18 |
| DM |  |  | 0.2 |  |  |  | 0.063 |
| No | 854 (80%) | 59 (73%) |  |  | 2578 (76%) | 226 (70%) |  |
| Diet | 30 (2.8%) | 3 (3.7%) |  |  | 126 (3.7%) | 10 (3.1%) |  |
| On Oral antidiabetics | 103 (10%) | 8 (10%) |  |  | 414 (12%) | 49 (15%) |  |
| Insulin | 81 (7.6%) | 11 (14%) |  |  | 279 (8.2%) | 37 (11%) |  |
| Hypertension | 814 (76%) | 64 (79%) | 0.7 |  | 2704 (80%) | 243 (75%) | 0.085 |
| Hypercholesteremia | 579 (54%) | 44 (54%) | 1 |  | 2180 (64%) | 193 (60%) | 0.15 |
| Current Smoker | 194 (18%) | 21 (26%) | 0.1 |  | 756 (22%) | 89 (28%) | 0.031 |
| Peripheral artery disease | 104 (10%) | 9 (11%) | 0.7 |  | 343 (10%) | 34 (11%) | 0.8 |
| Preoperative Stroke | 106 (10%) | 7 (8.6%) | 0.9 |  | 319 (9.4%) | 41 (13%) | 0.060 |
| Renal disease | 49 (4.6%) | 2 (2.5%) | 0.6 |  | 206 (6.1%) | 35 (11%) | 0.002 |
| Last pre-operative creatinine, µmol/l | 72 (61 to 88) | 73 (62 to 91) | 0.6 |  | 85 (74 to 100) | 89 (76 to 108) | 0.005 |
| Dialysis | 10 (0.94%) | 1 (1.2%) | 0.6 |  | 39 (1.1%) | 6 (1.9%) | 0.3 |
| COPD | 119 (11%) | 10 (12%) | 0.7 |  | 363 (11%) | 37 (11%) | 0.6 |
| Prior MI | 241 (23%) | 24 (30%) | 0.17 |  | 1250 (37%) | 121 (38%) | 0.8 |
| 3-Vessel CAD | 310 (29%) | 24 (30%) | 0.9 |  | 1746 (51%) | 151 (47%) | 0.13 |
| Main stem CAD | 85 (8.0%) | 8 (10%) | 0.5 |  | 528 (16%) | 45 (14%) | 0.5 |
| NYHA |  |  | 0.028 |  |  |  | 0.7 |
| n/a | 76 (7.1%) | 5 (6.2%) |  |  | 396 (12%) | 29 (9.0%) |  |
| I | 174 (16%) | 13 (16%) |  |  | 868 (26%) | 89 (28%) |  |
| II | 374 (35%) | 16 (20%) |  |  | 1196 (35%) | 116 (36%) |  |
| III | 386 (36%) | 40 (49%) |  |  | 767 (23%) | 73 (23%) |  |
| IV | 58 (5.4%) | 7 (8.6%) |  |  | 170 (5.0%) | 15 (4.7%) |  |
| NYHA III or IV | 444 (42%) | 47 (58%) | 0.005 |  | 937 (28%) | 88 (27%) | 1 |
| AF preoperative | 136 (13%) | 10 (12%) | 1 |  | 350 (10%) | 44 (14%) | 0.071 |
| Ejection fraction | 60 (51 to 65) | 58 (50 to 63) | 0.3 |  | 56 (48 to 60) | 56 (45 to 62) | 0.9 |
| EuroSCORE II | 2.8(1.5 to 5.6) | 3.0(1.7 to 7.5) | 0.18 |  | 1.8(0.99 to 3.7) | 2.0 (1.1 to 4.3) | 0.064 |
| *BMI* Body Mass Index; *DM* Diabetes Mellitus; *COPD* Chronic Obstructive Pulmonary Disorder; *MI* Myocardial Infarction; *CAD* Coronary Artery Disease; *NYHA* New York Heart Association; *AF* Atrial Fibrillation | | | | | | | |

## Table 4 Predictors of readmission, adjusted incidence rate ration

| Analysis | Variables | IRR (95%) | p |
| --- | --- | --- | --- |
| Simple adjustment | Female | 0.82 (0.64 to 1.05) | 0.11 |
|  | Age | 0.99 (0.98 to 1.00) | 0.068 |
|  | Female | 0.80 (0.63 to 1.03) | 0.078 |
|  | COPD | 1.03 (0.76 to 1.41) | 0.8 |
|  | Female | 0.78 (0.61 to 1.00) | 0.051 |
|  | Euroscore2 | 1.02 (1.00 to 1.03) | 0.007 |
|  | Female | 0.80 (0.62 to 1.02) | 0.070 |
|  | Emergency | 0.63 (0.39 to 1.03) | 0.068 |
|  | Female | 0.79 (0.62 to 1.01) | 0.063 |
|  | Any CABG | 0.93 (0.76 to 1.14) | 0.5 |
|  | Female | 0.80 (0.63 to 1.03) | 0.082 |
|  | Any AV | 0.97 (0.79 to 1.20) | 0.8 |
|  | Female | 0.77 (0.61 to 0.99) | 0.042 |
|  | Any MV | 1.34 (1.06 to 1.69) | 0.013 |
|  | Female | 0.80 (0.63 to 1.03) | 0.080 |
|  | Thoracic aorta | 1.10 (0.81 to 1.50) | 0.5 |
|  | Female | 0.82 (0.63 to 1.06) | 0.12 |
|  | ECC time, per 10 min | 1.04 (1.02 to 1.06) | <0.001 |
| Interaction terms | Female and Age | 1.00 (0.98 to 1.02) | 0.8 |
|  | Female and Euroscore2 | 0.98 (0.95 to 1.01) | 0.11 |
|  | Female and ECC time, per 10 min | 0.99 (0.95 to 1.04) | 0.7 |
|  | Female and COPD | 1.10 (0.52 to 2.35) | 0.8 |
|  | Female and Emergency | 1.09 (0.29 to 4.05) | 0.9 |
|  | Female and any CABG | 1.48 (0.90 to 2.43) | 0.12 |
|  | Female and any AV | 1.17 (0.71 to 1.93) | 0.5 |
|  | Female and any MV | 0.66 (0.38 to 1.14) | 0.14 |
|  | Female and Thoracic aorta | 0.51 (0.20 to 1.28) | 0.15 |
| Adjustment for surgery | Female | 0.78 (0.61 to 1.00) | 0.052 |
|  | Any CABG | 1.07 (0.82 to 1.39) | 0.6 |
|  | Any AV | 1.03 (0.80 to 1.32) | 0.8 |
|  | Any MV | 1.41 (1.07 to 1.85) | 0.015 |
|  | Thoracic aorta | 1.17 (0.84 to 1.64) | 0.3 |
| Full adjustment | Female | 0.78 (0.60 to 1.02) | 0.071 |
|  | Age | 0.99 (0.98 to 1.00) | 0.051 |
|  | COPD | 1.01 (0.72 to 1.41) | 1 |
|  | EuroSCORE2 | 1.02 (1.01 to 1.04) | 0.002 |
|  | emergency | 0.42 (0.23 to 0.75) | 0.004 |
|  | Any CABG | 1.01 (0.76 to 1.34) | 1 |
|  | Any AV | 0.88 (0.67 to 1.16) | 0.4 |
|  | Any MV | 1.10 (0.79 to 1.53) | 0.6 |
|  | Thoracic aorta | 0.89 (0.61 to 1.30) | 0.6 |
|  | ECC time, per 10 min | 1.02 (1.00 to 1.05) | 0.066 |

## Table 5 Incidence Rate Ratios of Postoperative Complications

| Analysis | Variables | IRR (95% CI) | p |
| --- | --- | --- | --- |
| Crude | Length of ICU stay, days | 1.01 (1.00 to 1.03) | 0.090 |
|  | Intubation >72h | 1.64 (0.97 to 2.80) | 0.067 |
|  | Reoperation for bleeding | 1.88 (1.27 to 2.80) | 0.002 |
|  | Postoperative MI | 1.13 (0.53 to 2.39) | 0.747 |
|  | Postoperative Stroke | 1.47 (0.93 to 2.32) | 0.099 |
|  | AF at discharge | 1.24 (1.00 to 1.53) | 0.047 |
|  | Permanent pacemaker | 1.01 (0.65 to 1.58) | 0.972 |
|  | Sternal infection | 5.35 (3.74 to 7.65) | <0.001 |
|  | Postoperative renal failure | 1.50 (0.97 to 2.31) | 0.065 |
|  | Renal substitution therapy | 1.46 (0.68 to 3.12) | 0.333 |
|  | Pulmonary infection | 1.19 (0.82 to 1.73) | 0.361 |
|  | MACCE | 1.40 (0.93 to 2.09) | 0.103 |
|  | Sepsis | 2.83 (1.67 to 4.81) | <0.001 |
|  | Length of stay | 1.00 (1.00 to 1.01) | 0.147 |
| Simple adjustment | Female | 0.80 (0.62 to 1.03) | 0.079 |
|  | Length of ICU stay, days | 1.01 (1.00 to 1.03) | 0.083 |
|  | Female | 0.80 (0.63 to 1.02) | 0.073 |
|  | Intubation >72h | 1.66 (0.98 to 2.83) | 0.061 |
|  | Female | 0.80 (0.63 to 1.02) | 0.077 |
|  | Reoperation for bleeding | 1.89 (1.27 to 2.81) | 0.002 |
|  | Female | 0.80 (0.63 to 1.02) | 0.078 |
|  | Postoperative MI | 1.14 (0.54 to 2.40) | 0.738 |
|  | Female | 0.80 (0.63 to 1.02) | 0.073 |
|  | Postoperative Stroke | 1.48 (0.94 to 2.34) | 0.091 |
|  | Female | 0.79 (0.62 to 1.01) | 0.065 |
|  | AF at discharge | 1.25 (1.01 to 1.55) | 0.039 |
|  | Female | 0.80 (0.63 to 1.03) | 0.078 |
|  | Permanent pacemaker | 1.02 (0.65 to 1.59) | 0.945 |
|  | Female | 0.76 (0.60 to 0.97) | 0.030 |
|  | Sternal infection | 5.49 (3.84 to 7.86) | <0.001 |
|  | Female | 0.80 (0.63 to 1.02) | 0.075 |
|  | Postoperative renal failure | 1.51 (0.98 to 2.32) | 0.062 |
|  | Female | 0.80 (0.63 to 1.03) | 0.078 |
|  | Renal substitution therapy | 1.46 (0.68 to 3.12) | 0.333 |
|  | Female | 0.80 (0.63 to 1.03) | 0.081 |
|  | Pulmonary infection | 1.18 (0.81 to 1.72) | 0.379 |
|  | Female | 0.80 (0.63 to 1.02) | 0.074 |
|  | MACCE | 1.41 (0.94 to 2.10) | 0.097 |
|  | Female | 0.81 (0.63 to 1.03) | 0.087 |
|  | Sepsis | 2.80 (1.65 to 4.76) | <0.001 |
|  | Female | 0.80 (0.63 to 1.03) | 0.081 |
|  | Length of stay | 1.00 (1.00 to 1.01) | 0.146 |

## Table 6 Baseline characteristics of patients undergoing CABG surgery by gender

|  | Total (N = 2005) | Male (N = 1700) | Female (N = 305) | p |
| --- | --- | --- | --- | --- |
| Age, years | 67 (60 to 73) | 66 (59 to 73) | 71 (64 to 76) | <0.001 |
| BMI, kg/m² | 27 (25 to 30) | 27 (25 to 30) | 27 (24 to 30) | 0.4 |
| DM |  |  |  | 0.4 |
| No | 1342 (67%) | 1141 (67%) | 201 (66%) |  |
| Diet | 96 (4.8%) | 81 (4.8%) | 15 (4.9%) |  |
| On oral antidiabetics | 312 (16%) | 270 (16%) | 42 (14%) |  |
| Insulin | 255 (13%) | 208 (12%) | 47 (15%) |  |
| Hypertension | 1749 (87%) | 1470 (86%) | 279 (91%) | 0.015 |
| Hypercholesteremia | 1549 (77%) | 1316 (77%) | 233 (76%) | 0.7 |
| Current smoker | 520 (26%) | 451 (27%) | 69 (23%) | 0.16 |
| Peripheral artery disease | 266 (13%) | 228 (13%) | 38 (12%) | 0.7 |
| Preoperative stroke | 162 (8.1%) | 132 (7.8%) | 30 (10%) | 0.3 |
| Renal disease | 97 (4.8%) | 85 (5.0%) | 12 (3.9%) | 0.6 |
| Last pre-operative creatinine, µmol/l | 81 (70 to 96) | 83 (73 to 98) | 69 (59 to 85) | <0.001 |
| Dialysis | 15 (0.75%) | 13 (0.76%) | 2 (0.66%) | 1 |
| COPD | 203 (10%) | 169 (10%) | 34 (11%) | 0.5 |
| Prior MI | 1186 (59%) | 1005 (59%) | 181 (59%) | 1 |
| Three vessel CAD | 1697 (85%) | 1457 (86%) | 240 (79%) | 0.002 |
| Main stem CAD | 527 (26%) | 454 (27%) | 73 (24%) | 0.3 |
| NYHA |  |  |  | <0.001 |
| n/a | 244 (12%) | 209 (12%) | 35 (11%) |  |
| I | 610 (30%) | 543 (32%) | 67 (22%) |  |
| II | 683 (34%) | 582 (34%) | 101 (33%) |  |
| III | 375 (19%) | 290 (17%) | 85 (28%) |  |
| IV | 93 (4.6%) | 76 (4.5%) | 17 (5.6%) |  |
| NYHA III or IV | 468 (23%) | 366 (22%) | 102 (33%) | <0.001 |
| AF preoperative | 98 (4.9%) | 89 (5.2%) | 9 (3.0%) | 0.11 |
| Ejection fraction | 55 (47 to 60) | 55 (46 to 60) | 58 (50 to 62) | 0.001 |
| EuroSCORE II | 1.5 (0.89 to 2.7) | 1.4 (0.85 to 2.5) | 2.2 (1.4 to 3.8) | <0.001 |

*BMI* Body Mass Index; *DM* Diabetes Mellitus; *COPD* Chronic Obstructive Pulmonary Disorder; *MI* Myocardial Infarction; *CAD* Coronary Artery Disease; *NYHA* New York Heart Association; *AF* Atrial Fibrillation

## Table 7 Subgroup analysis of patients after CABG surgery

|  | Total (N = 2005) | Male (N = 1700) | Female (N = 305) | P |
| --- | --- | --- | --- | --- |
| Readmission | 161 (8.0%) | 122 (7.2%) | 20 (6.6%) | 0.051 |
| Cardiac related | 48 (30%) | 44 (32%) | 4 (18%) | 0.3 |
| Infection | 44 (27%) | 32 (23%) | 12 (55%) | 0.004 |
| Bleeding | 18 (11%) | 18 (13%) | 0 (0.00%) | 0.14 |
| Neurological | 7 (4.3%) | 6 (4.3%) | 1 (4.5%) | 1 |
| Non infectious wound complications | 12 (7.5%) | 8 (5.8%) | 4 (18%) | 0.062 |
| Gastrointestinal disease | 5 (3.1%) | 5 (3.6%) | 0 (0.00%) | 1 |
| Pain excluding angina | 14 (8.7%) | 13 (9.4%) | 1 (4.5%) | 0.7 |
| Musculoskeletal disorders | 6 (3.7%) | 6 (4.3%) | 0 (0.00%) | 1 |
| Urological disorders | 3 (1.9%) | 3 (2.2%) | 0 (0.00%) | 1 |
| Other reasons | 4 (2.5%) | 4 (2.9%) | 0 (0.00%) | 1 |

*derived from incidence rate ratio.

Note that numbers in the first row relate to patients, the other rows refer to readmissions.

## Table 8 Subgroup analysis of patient undergoing isolated aortic valve surgery

|  | Total (N = 695) | Male (N = 452) | Female (N = 243) | p |
| --- | --- | --- | --- | --- |
| Age, years | 71 (62 to 77) | 70 (60 to 77) | 73 (65 to 78) | 0.001 |
| BMI, kg/m² | 26 (23 to 30) | 27 (24 to 29) | 25 (22 to 30) | 0.10 |
| Diabetes Mellitus |  |  |  | 0.60 |
| No | 561 (81%) | 360 (80%) | 201 (83%) |  |
| Diet | 18 (2.6%) | 13 (2.9%) | 5 (2.1%) |  |
| On Oral antidiabethics | 74 (11%) | 48 (11%) | 26 (11%) |  |
| Insulin | 42 (6.0%) | 31 (6.9%) | 11 (4.5%) |  |
| Hypertension | 494 (71%) | 318 (70%) | 176 (72%) | 0.60 |
| Hypercholesteremia | 377 (54%) | 257 (57%) | 120 (49%) | 0.07 |
| Current Smoker | 144 (21%) | 102 (23%) | 42 (17%) | 0.12 |
| Peripheral artery disease | 77 (11%) | 49 (11%) | 28 (12%) | 0.80 |
| Preoperative Stroke | 63 (9.1%) | 43 (10%) | 20 (8.2%) | 0.68 |
| Renal disease | 53 (7.6%) | 43 (10%) | 10 (4.1%) | 0.011 |
| Last pre-operative creatinine, µmol/l | 81 (68 to 101) | 87 (75 to 109) | 69 (60 to 85) | <0.001 |
| Dialysis | 12 (1.7%) | 8 (1.8%) | 4 (1.6%) | 1.00 |
| COPD | 93 (13%) | 62 (14%) | 31 (13%) | 0.82 |
| Prior MI | 52 (7.5%) | 41 (9.1%) | 11 (4.5%) | 0.034 |
| Three vessel CAD | 0 (0%) | 0 (0%) | 0 (0%) |  |
| Left main CAD | 0 (0%) | 0 (0%) | 0 (0%) |  |
| NYHA |  |  |  | 0.004 |
| n/a | 36 (5.2%) | 27 (6.0%) | 9 (3.7%) |  |
| I | 97 (14%) | 76 (17%) | 21 (8.6%) |  |
| II | 254 (37%) | 167 (37%) | 87 (36%) |  |
| III | 270 (39%) | 162 (36%) | 108 (44%) |  |
| IV | 38 (5.5%) | 20 (4.4%) | 18 (7.4%) |  |
| NYHA III or IV | 308 (44%) | 182 (40%) | 126 (52%) | 0.004 |
| AF preop | 79 (11%) | 54 (12%) | 25 (10%) | 0.53 |
| Ejection fraction | 60 (50 to 64) | 57 (48 to 63) | 60 (55 to 65) | <0.001 |
| Euroscore2 | 1.6 (0.98 to 3.6) | 1.4 (0.88 to 3.1) | 2.1 (1.2 to 4.1) | <0.001 |
| *BMI* Body Mass Index; *DM* Diabetes Mellitus; *COPD* Chronic Obstructive Pulmonary Disorder; *MI* Myocardial Infarction; *CAD* Coronary Artery Disease; *NYHA* New York Heart Association; *AF* Atrial Fibrillation | | | | |

## Table 9 Reasons for readmission in isolated aortic valve surgery patients

|  | Total (N = 695) | Male (N = 452) | Female (N = 243) | P |
| --- | --- | --- | --- | --- |
| Overall readmission | 58 (8.3%) | 42 (9.3%) | 16 (6.6%) | 0.28* |
| Cardiac related | 15 (26%) | 11 (26%) | 4 (25%) | 1.00 |
| Infection | 15 (26%) | 11 (26%) | 4 (25%) | 1.00 |
| Bleeding | 8 (14%) | 4 (10%) | 4 (25%) | 0.20 |
| Neurological | 3 (5.2%) | 1 (2.4%) | 2 (13%) | 0.18 |
| Non infectious wound complications | 2 (3.4%) | 0 (0%) | 2 (13%) | 0.07 |
| Gastrointestinal disease (no bleeding) | 2 (3.4%) | 2 (4.8%) | 0 (0%) | 1.00 |
| Pain excluding angina | 4 (6.9%) | 4 (10%) | 0 (0%) | 0.57 |
| Musculoskeletal disorders | 3 (5.2%) | 3 (7.1%) | 0 (0%) | 0.55 |
| Urological disorders | 1 (1.7%) | 1 (2.4%) | 0 (0%) | 1.00 |
| Other reasons | 0 (0%) |  |  | 1.00 |

*derived from incidence rate ratio.

Note that numbers in the first row relate to patients, the other rows refer to readmissions.

## Table 10 Subgroup analysis of patient undergoing isolated mitral valve surgery

|  | N | Total (N = 464) | Male (N = 283) | Female (N = 181) | P |
| --- | --- | --- | --- | --- | --- |
| Age, years | 464 | 63 (54 to 72) | 62 (53 to 70) | 66 (57 to 73) | 0.005 |
| BMI, kg/m² | 464 | 25 (22 to 28) | 26 (23 to 28) | 23 (21 to 28) | <0.001 |
| Diabetes Mellitus | 464 |  |  |  | 0.53 |
| No |  | 427 (92%) | 258 (91%) | 169 (93%) |  |
| Diet |  | 5 (1.1%) | 4 (1.4%) | 1 (0.55%) |  |
| On Oral antidiabethics |  | 19 (4.1%) | 14 (4.9%) | 5 (2.8%) |  |
| Insulin |  | 13 (2.8%) | 7 (2.5%) | 6 (3.3%) |  |
| Hypertension | 464 | 276 (59%) | 167 (59%) | 109 (60%) | 0.85 |
| Hypercholesteremia | 464 | 145 (31%) | 84 (30%) | 61 (34%) | 0.41 |
| Current Smoker | 464 | 57 (12%) | 40 (14%) | 17 (9.4%) | 0.15 |
| Peripheral artery disease | 464 | 10 (2.2%) | 3 (1.1%) | 7 (3.9%) | 0.05 |
| Preoperative Stroke | 464 | 38 (8.2%) | 24 (8.5%) | 14 (7.7%) | 0.86 |
| Renal disease | 464 | 17 (3.7%) | 13 (4.6%) | 4 (2.2%) | 0.21 |
| Last pre-operative creatinine, µmol/l | 464 | 80 (69 to 96) | 84 (75 to 101) | 72 (62 to 84) | <0.001 |
| Dialysis | 464 | 2 (0.43%) | 2 (0.71%) | 0 (0.00%) | 0.52 |
| COPD | 464 | 38 (8.2%) | 24 (8.5%) | 14 (7.7%) | 0.86 |
| Prior MI | 464 | 20 (4.3%) | 15 (5.3%) | 5 (2.8%) | 0.24 |
| Three vessel CAD | 464 | 0 (0%) | 0 (0%) | 0 (0%) |  |
| Left main CAD | 464 | 0 (0%) | 0 (0%) | 0 (0%) |  |
| NYHA | 464 |  |  |  | <0.001 |
| n/a |  | 44 (9.5%) | 37 (13%) | 7 (3.9%) |  |
| I |  | 85 (18%) | 64 (23%) | 21 (12%) |  |
| II |  | 192 (41%) | 109 (39%) | 83 (46%) |  |
| III |  | 120 (26%) | 58 (20%) | 62 (34%) |  |
| IV |  | 23 (5.0%) | 15 (5.3%) | 8 (4.4%) |  |
| NYHA III or IV | 464 | 143 (31%) | 73 (26%) | 70 (39%) | 0.004 |
| AF preop | 464 | 88 (19%) | 51 (18%) | 37 (20%) | 0.54 |
| Ejection fraction | 464 | 60 (55 to 65) | 60 (55 to 65) | 60 (55 to 66) | 0.46 |
| Euroscore2 | 464 | 1.2 (0.75 to 2.1) | 0.97 (0.67 to 1.6) | 1.6 (1.0 to 2.7) | <0.001 |
| *BMI* Body Mass Index; *DM* Diabetes Mellitus; *COPD* Chronic Obstructive Pulmonary Disorder; *MI* Myocardial Infarction; *CAD* Coronary Artery Disease; *NYHA* New York Heart Association; *AF* Atrial Fibrillation | | | | | |

## Table 11 Reasons for readmission in isolated mitral valve surgery patients

|  | Total (N = 464) | Male (N = 283) | Female (N = 181) | P |
| --- | --- | --- | --- | --- |
| Overall readmission | 40 (8.6%) | 32 (11.3%) | 8 (4.46%) | 0.014* |
| Cardiac related | 10 (25%) | 7 (22%) | 3 (38%) | 0.39 |
| Infection | 10 (25%) | 9 (28%) | 1 (13%) | 0.65 |
| Bleeding | 6 (15%) | 5 (16%) | 1 (13%) | 1.00 |
| Neurological | 3 (7.5%) | 3 (9.4%) | 0 (0.00%) | 1.00 |
| Non infectious wound complications | 2 (5.0%) | 2 (6.3%) | 0 (0.00%) | 1.00 |
| Gastrointestinal disease (no bleeding) | 3 (7.5%) | 2 (6.3%) | 1 (13%) | 0.50 |
| Pain excluding angina | 2 (5.0%) | 2 (6.3%) | 0 (0.00%) | 1.00 |
| Musculoskeletal disorders | 1 (2.5%) | 0 (0.00%) | 1 (13%) | 0.20 |
| Other reasons | 3 (7.5%) | 2 (6.3%) | 1 (13%) | 0.50 |

*derived from incidence rate ratio.

Note that numbers in the first row relate to patients, the other rows refer to readmissions.

## Table 12 Outpatient versus inpatient Rehabilitation in 1646 patients with available information

|  | Total (N = 1462) | Outpatient rehab  (N = 263) | Inpatient rehab  (N = 1199) | p |
| --- | --- | --- | --- | --- |
| Age, years | 68 (60 to 74) | 63 (56 to 70) | 69 (61 to 75) | <0.001 |
| BMI, kg/m² | 27 (24 to 29) | 26 (24 to 29) | 27 (24 to 29) | 0.090 |
| DM |  |  |  | 0.5 |
| No | 1075 (74%) | 203 (77%) | 872 (73%) |  |
| Diet | 75 (5.1%) | 10 (3.8%) | 65 (5.4%) |  |
| On oral antidiabetics | 184 (13%) | 29 (11%) | 155 (13%) |  |
| Insulin | 128 (8.8%) | 21 (8.0%) | 107 (8.9%) |  |
| Hypertension | 1084 (74%) | 187 (71%) | 897 (75%) | 0.2 |
| Hypercholesteremia | 818 (56%) | 158 (60%) | 660 (55%) | 0.15 |
| Current smoker | 311 (21%) | 63 (24%) | 248 (21%) | 0.2 |
| Peripheral artery disease | 137 (9.4%) | 16 (6.1%) | 121 (10%) | 0.047 |
| Preoperative stroke | 147 (10%) | 20 (7.6%) | 127 (11%) | 0.17 |
| Renal disease | 77 (5.3%) | 7 (2.7%) | 70 (5.8%) | 0.033 |
| Last pre-operative creatinine, µmol/l | 84 (70 to 98) | 82 (69 to 94) | 84 (71 to 99) | 0.18 |
| Dialysis | 8 (0.55%) | 1 (0.38%) | 7 (0.58%) | 1 |
| COPD | 132 (9.0%) | 20 (7.6%) | 112 (9.3%) | 0.4 |
| Prior MI | 386 (26%) | 81 (31%) | 305 (25%) | 0.076 |
| Three vessel CAD | 668 (46%) | 112 (43%) | 556 (46%) | 0.3 |
| Main stem coronary artery disease | 159 (11%) | 31 (12%) | 128 (11%) | 0.6 |
| NYHA III or IV | 319 (22%) | 35 (13%) | 284 (24%) | <0.001 |
| AF preoperative | 139 (10%) | 15 (5.7%) | 124 (10%) | 0.020 |
| Ejection fraction, % | 59 (50 to 63) | 59 (50 to 64) | 58 (50 to 63) | 0.7 |
| Euroscore II | 1.8 (1.1 to 3.8) | 1.3 (0.79 to 2.4) | 2.0 (1.1 to 4.2) | <0.001 |
| *BMI* Body Mass Index; *DM* Diabetes Mellitus; *COPD* Chronic Obstructive Pulmonary Disorder; *MI* Myocardial Infarction; *CAD* Coronary Artery Disease; *NYHA* New York Heart Association; *AF* Atrial Fibrillation; *Please note that 184 patients neither went to an outpatient nor inpatient rehabilitation.* | | | | |
